# Lipidomics-based tissue heterogeneity in specimens of luminal breast cancer revealed by clustering analysis of mass spectrometry imaging: A preliminary study

**DOI:** 10.1371/journal.pone.0283155

**Published:** 2023-05-10

**Authors:** Shuhei Aramaki, Shogo Tsuge, Ariful Islam, Fumihiro Eto, Takumi Sakamoto, Soho Oyama, Wenxin Li, Chi Zhang, Shinichi Yamaguchi, Daiki Takatsuka, Yuko Hosokawa, A. S. M. Waliullah, Yutaka Takahashi, Kenji Kikushima, Tomohito Sato, Kei Koizumi, Hiroyuki Ogura, Tomoaki Kahyo, Satoshi Baba, Norihiko Shiiya, Haruhiko Sugimura, Katsumasa Nakamura, Mitsutoshi Setou

**Affiliations:** 1 Department of Cellular and Molecular Anatomy, Hamamatsu University School of Medicine, Hamamatsu, Shizuoka, Japan; 2 Department of Radiation Oncology, Hamamatsu University School of Medicine, Hamamatsu, Shizuoka, Japan; 3 First Department of Pathology, Hamamatsu University School of Medicine, Hamamatsu, Shizuoka, Japan; 4 Analytical & Measuring Instruments Division, Shimadzu Corporation, Kyoto, Japan; 5 1st Department of Surgery, Hamamatsu University School of Medicine, Hamamatsu, Shizuoka, Japan; 6 International Mass Imaging Center, Hamamatsu University School of Medicine, Hamamatsu, Shizuoka, Japan; 7 Department of Diagnostic Pathology, Hamamatsu University School of Medicine, Hamamatsu, Japan; Kimura Hoospital, JAPAN

## Abstract

Cancer tissues reflect a greater number of pathological characteristics of cancer compared to cancer cells, so the evaluation of cancer tissues can be effective in determining cancer treatment strategies. Mass spectrometry imaging (MSI) can evaluate cancer tissues and even identify molecules while preserving spatial information. Cluster analysis of cancer tissues’ MSI data is currently used to evaluate the phenotype heterogeneity of the tissues. Interestingly, it has been reported that phenotype heterogeneity does not always coincide with genotype heterogeneity in HER2-positive breast cancer. We thus investigated the phenotype heterogeneity of luminal breast cancer, which is generally known to have few gene mutations. As a result, we identified phenotype heterogeneity based on lipidomics in luminal breast cancer tissues. Clusters were composed of phosphatidylcholine (PC), triglycerides (TG), phosphatidylethanolamine, sphingomyelin, and ceramide. It was found that mainly the proportion of PC and TG correlated with the proportion of cancer and stroma on HE images. Furthermore, the number of carbons in these lipid class varied from cluster to cluster. This was consistent with the fact that enzymes that synthesize long-chain fatty acids are increased through cancer metabolism. It was then thought that clusters containing PCs with high carbon counts might reflect high malignancy. These results indicate that lipidomics-based phenotype heterogeneity could potentially be used to classify cancer for which genetic analysis alone is insufficient for classification.

## Introduction

In vivo, cancer tissues are not simply cancer cells alone; rather, they are a mixture of cancer cells and surrounding cells. Although cellular and genomic analyses have been used to classify cancer cells in order to determine treatment strategies [1–4], cancer tissues have more complex characters than the cellular unit, and the ability to characterize cancer tissues in greater detail will enable more accurate cancer classification. Mass spectrometry imaging (MSI) is one of the techniques that can evaluate cancer tissues; it can identify and visualize molecules on tissue sections while maintaining the molecules’ positional information. MSI is also suitable for measuring the molecular weight of lipids and fatty acids, and MSI has shown that the expression of certain lipids and fatty acids is enhanced in cancer tissues, unlike normal tissues [5,6].

Lipid molecules are very diverse due to their vast combinations of carbons double bonds, and the wide variety of expressions of these lipid molecules plays an important role in reflecting the complex pathology of cancer tissues [7,8]. It has been reported that multivariate grouping (by a cluster analysis) of these data may be able to describe the heterogeneity of cancer tissues [9].

In breast cancer, the subtype determined by immunohistochemistry of estrogen receptor (ER), progesterone receptor (PgR), and human epidermal growth factor receptor type 2 (HER2) is one of the prognostic factors as well as a factor in the clinical stage, histological grade, and the marker Ki-67 [10–12]. The triple-negative (ER-, PgR-, and HER2-negative) subtype of breast cancer was reported to have a poor prognosis compared to luminal (ER-positive and/or PgR-negative and HER2-negative) breast cancer [11]. Additional prognostic factors among these subtypes are known, such as the recurrence score of Oncotype DX for luminal breast cancer [13]. It has also been suggested that changes in the metabolism of a wide variety of lipids in breast cancer (including phosphatidylcholine, phosphatidylethanolamine, sphingomyelin, and triglycerides) are associated with the tumor grade and prognosis [6,7].

We conducted the present study to investigate whether a cluster analysis of MSI data can represent heterogeneity in luminal breast cancer tissues based on the type of lipid molecules and their expression levels (lipidomics).

## Materials and methods

### Clinical materials

We collected tissue samples of 12 cases of invasive breast cancer surgeries from among the surgeries performed at Hamamatsu University School of Medicine between February 2013 and May 2017. Tissues of the primary lesions were collected at the time of surgery. All of the patients were Japanese women who were aged 42–69 years (mean age, 54 yrs). The primary lesions were histologically diagnosed as breast cancer by experienced pathologists. The tumor-node-metastasis (TNM) classifications were T1bN0M0–T2N0M0 (Stages 1–2A). The breast cancer specimens were all positive for immunohistochemical staining of estrogen receptor and progesterone receptor and negative for immunohistochemical staining of HER2.

The collected human breast cancer specimens were immediately frozen by liquid N2. All specimens were kept in a cryostat chamber (CM1950; Leica, Wetzler, Germany) at −20°C for approx. 30 min just before cryo-sectioning. Thereafter, 10-μm-thick sections were cut from the unfixed tissue specimens and mounted on uncoated glass slides (Matsunami, Osaka, Japan) for desorption electrospray ionisation (DESI)-MSI measurement. The same specimens were stained with hematoxylin and eosin stain (H&E) after MSI measurement. Based on the findings of the H&E-stained images, three of the 12 tissues were diagnosed as inadequate for evaluation in this study, and the remaining nine patients’ tissues were included in the analysis.

### Breast cancer subtypes (luminal A and luminal B)

In this study, we defined ’luminal A’ as ER+ and/or PgR+, Ki-67 low and HER2-, and ’luminal B’ as ER+ and/or PgR+, Ki-67 high and HER2-. The samples with low Ki-67 values (<14%) are classified as luminal A, and those with high Ki-67 values were classified as luminal B. The 14% cut-off point for the Ki-67 values is derived from a comparison with gene array data as a prognostic factor [11].

### DESI-MSI

In this study, DESI-MSI was used as the MSI technique. DESI-MSI does not require tissue pretreatment and can be performed at atmospheric pressure. It also provides the soft ionization and visualization of molecules in their original state. DESI-MSI data were acquired in positive ion mode at *m/z* range 100–1100 on a quadrupole time-of-flight mass spectrometer (Xevo G2-XS, Waters, Milford, MA, USA). The mass spectrometer was first calibrated with 500 mM sodium formate dissolved in 2-propanol: water (90:10, v/v) prior to measurement. The spray solvent (98% MeOH, 2% water) was delivered at 3 μL/min using a solvent pump. In this study, all samples were measured at the same time (4 March 2018). In this case, the results are not biased by the experimental equipment or measurement conditions, but the storage time of the samples may affect the results.

Optimization of the DESI ionization source was performed to obtain the maximum signal intensity from the tissues. using the following parameters: capillary voltage: 4.0 kV, source temperature: 120°C, nebulizing gas (nitrogen gas) pressure: 0.4 MPa, incidence angle of sprayer: 80°, inlet to sprayer distance: ~5 mm, inlet to surface distance: ~0.5 mm, and surface to sprayer distance: ~2 mm. After the optimization of the DESI source, samples were placed on the 2D stage and the data were acquired using the following parameters: scan speed: 300 μm/sec, pixel size: 100 μm, mass resolution: 22000, and mass window: 0.02 Da. The limit of detection of DESI was 1 μg/ml for phosphatidylcholine (16:00/16:00).

### Data import and preprocess

The DESI-MSI data were acquired with MassLynx ver. 4.1 (Waters) and processed by HDImaging ver. 1.4 (Waters). All data were normalized by the total ion current (TIC) and converted to imzml data using the HDImaging software. Then, the imzml data were converted to IMDX format using the IMDX converter ver. 1.12.0.10258 (Shimadzu, Kyoto, Japan). The data were processed using the centroid method. Mass correction was processed with *m/z* 309.2036 ([C_16_H_30_O_4_+Na]^+^) as a lock mass. This ion is detected daily as the background ion by our equipment. In the present case, this ion was detected as *m/z* 309.20303. The peak width was set up as 50 ppm. The converted data were imported into IMAGEREVEAL™ MS ver. 1.3 (Shimadzu) for further analysis.

### Dimensionality reduction

We performed dimensionality reduction before K-means clustering. The most common method of dimensionality reduction is a principal component analysis (PCA) [14], but a nonlinear dimensionality-reduction technique, i.e., uniform manifold approximation and projection (UMAP) has recently been used [15]. In the present study, we performed dimensionality reduction with UMAP with the following parameters: n_neighbors  =  5, n_components  =  3, random_state  =  42 [16].

### Multivariate analysis

K-means (for a non-hierarchical cluster analysis) was selected as the multivariate analysis mode [17,18]. K-means was performed with the number of clusters from 2 to 15. We evaluated the clustering by creating silhouette plots (python 3.10, scikit-learn 1.2.1) to check the validity of the clustering analysis in the range of 2 to 15 clusters (S1 Fig). Among clusters 2 to 15, peak silhouette coefficients were found at 4 and 13. Since the aim of this study was to evaluate the heterogeneity of the cancer tissue and to verify whether it could be applied clinically as a preliminary study, the number of clusters was set at 13 by comparison with HE-stained images. As shown in S2 Fig, the selection of 13 clusters confirms that even detailed pathological findings can be reflected.

### Partial least squares (PLS)

We used the above-described IMAGEREVEAL™ MS software to perform a linear regression analysis, i.e., the partial least squares (PLS) method (also known as partial least squares regression). The PLS method extracts principal components that are related to the dependent variable in high-dimensional data. We set ROIs on the clusters. The PLS parameters were set as 1 (Y-value) ROI and 0 (Y-value) for the area excluding the ROI. In the present study, we focused on the 30 molecules with high PLS coefficients; since MS/MS measurements were not performed, we evaluated the 30 molecules whose expression matched those known to be upregulated in breast cancer in the previous reports [7,19–24].

### Ethics statement

All procedures were carried out following relevant national and international guidelines, including the Declaration of Helsinki in its present form. This study was approved by the Ethics Committee of the Hamamatsu University School of Medicine (project identification code: 14–114). All patients were provided with sufficient information, and a written informed consent for their materials to be used was obtained from all patients before their surgery. All patients were recruited to the study between February 2013 and May 2017.

### Data confidentiality

Only the authors affiliated with 1st Department of Surgery, Hamamatsu University School of Medicine have access to patient-identifiable information. The patient identifiable code table is stored in an environment that is not connected to the Internet.

## Results

**Fig 1:**
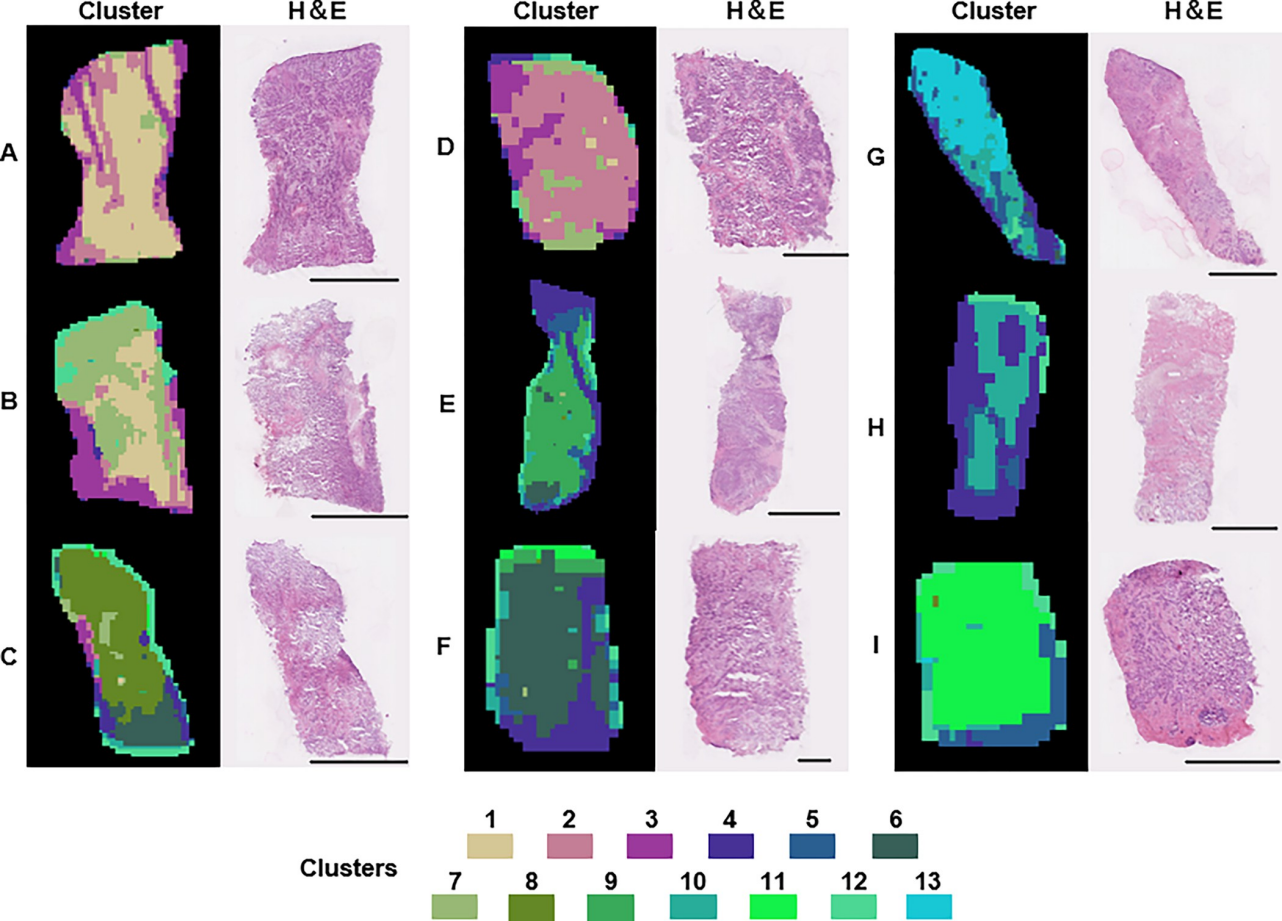
The clustering of the DESI-MSI data of the breast cancer tissues revealed differing heterogeneity in the luminal subtype. The cluster image and H&E images of nine breast cancer tissues are shown. The cluster image consists of 13 color clusters. The similarity of colors has nothing to do with the nature of the cluster. The lengths of the scale bars are as follows. A: 2500 μm, B: 2500 μm, C: 2500 μm, D: 1250 μm, E: 2500 μm, F: 500 μm, G: 2500 μm, H: 1250 μm, and I: 1250 μm.

**Fig 2:**
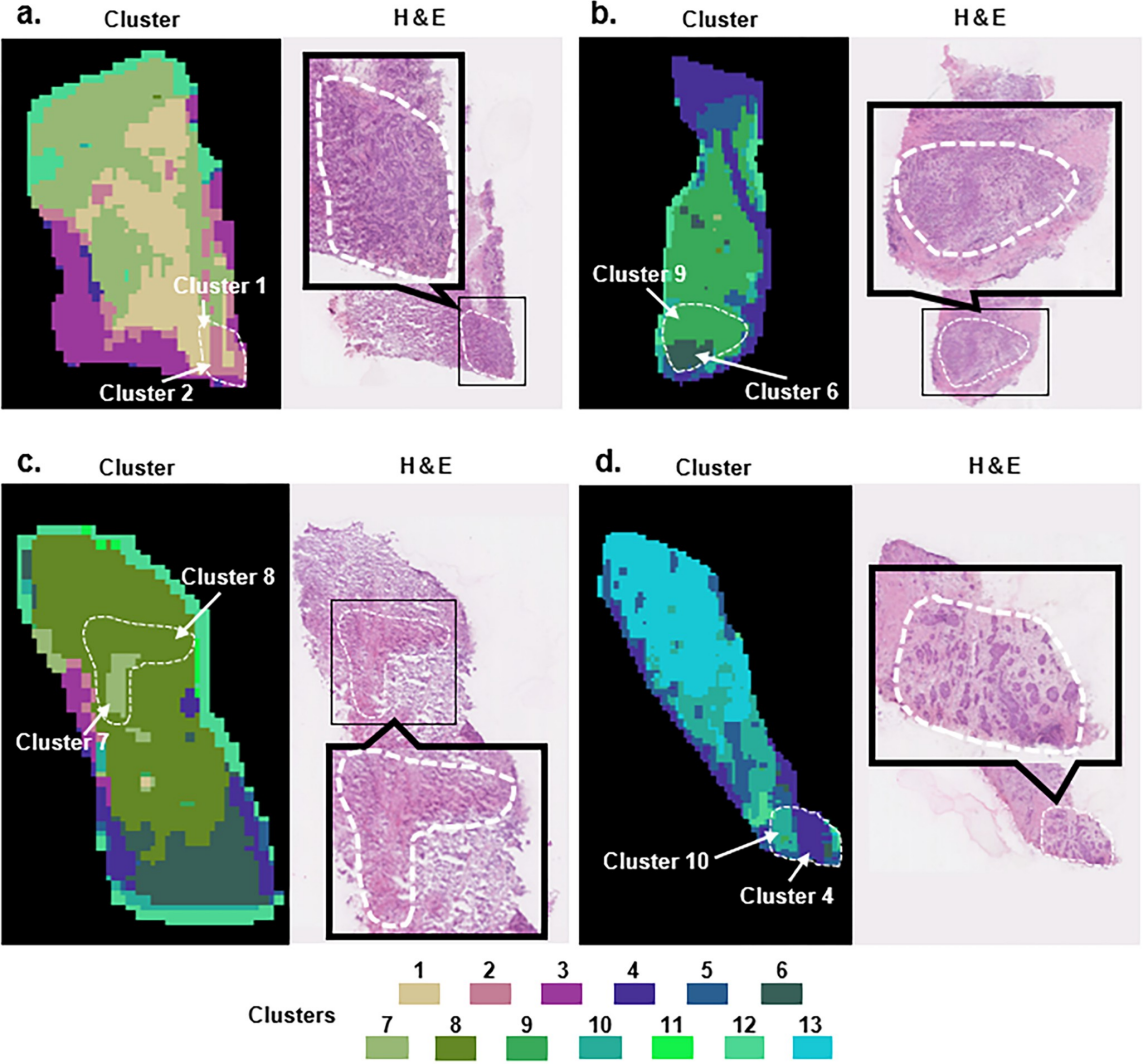
Examples of intra-tumor heterogeneity expressed by lipidomics-based clusters. The cancerous areas are circled with a *white dotted line*. **a:** The cancerous area is classified by clusters 1 and 2. **b:** The cancerous area is classified by clusters 6 and 9. **c:** The cancerous area is classified by clusters 7 and 8. **d:** The cancerous area is classified by clusters 4 and 10.

The results of the cluster analysis of the MSI data from the nine luminal breast cancer cases are presented in Fig 1. We found out that the lipidomics-based clusters were not only consistent with H&E findings, but also exhibited intra-tumor heterogeneity. Despite similar pathological findings on HE-stained images, the findings were classified into different clusters (Fig 2). In Fig 2A, heterogeneity in cancerous tissue by clusters 1 and 2 was observed. Fig 2B also shows heterogeneity in cancerous tissue by clusters 6 and 9. Compared to Fig 2A, the cancerous tissue included stroma tissue in Fig 2B. Fig 2C shows heterogeneity in cancerous and stroma tissue by clusters 7 and 8. Fig 2D shows heterogeneity in cancerous tissue by clusters 4 and 10. Compared to Fig 2A, the glandular duct structure was preserved in Fig 2D. Thus, heterogeneity was represented by different clusters in cases of subtle differences in pathology.

**Fig 3:**
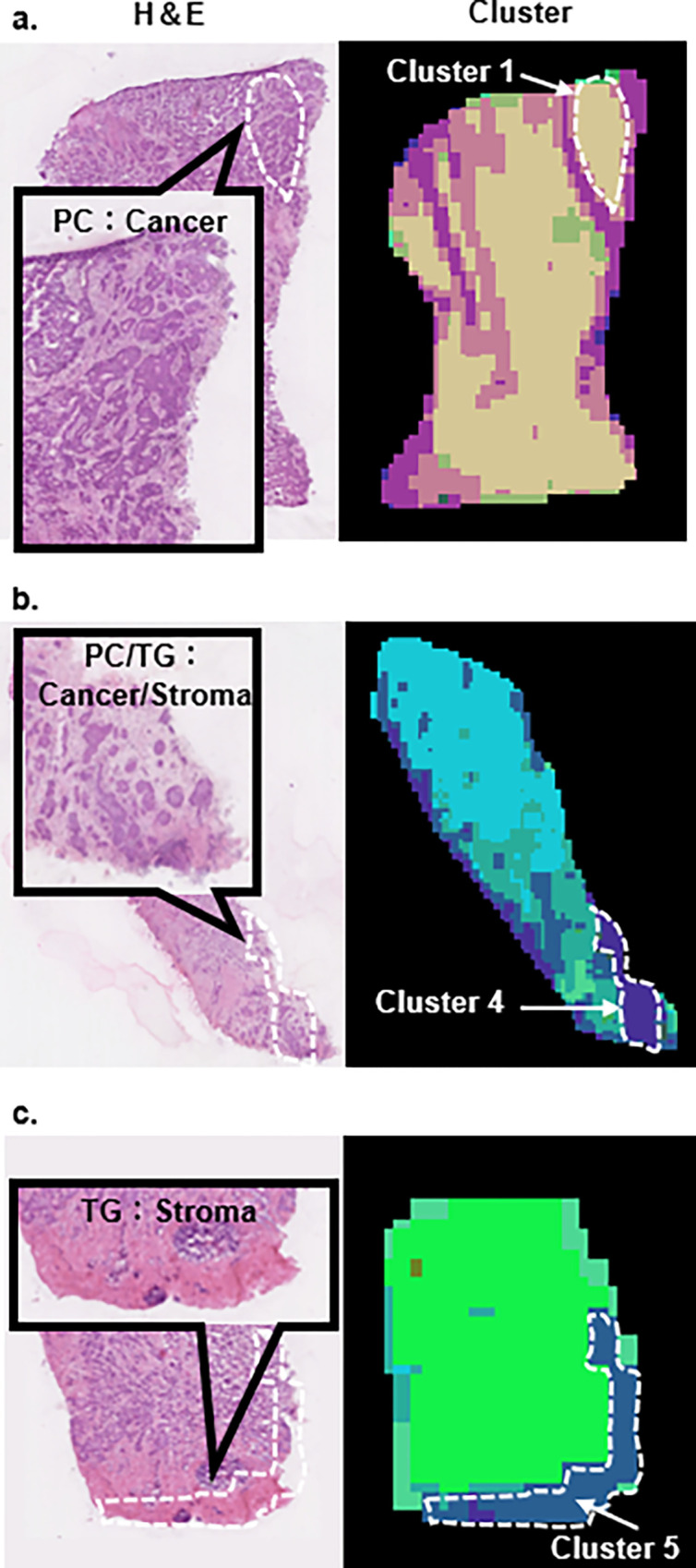
Representative findings of the HE-stained images corresponding to each lipid composition. a. Cluster 1, which is mainly composed of PC, is consistent with a dense finding of cancer cells in the HE stained images. b. Cluster 4, which is a mixture of PC and TG, is consistent with an equal mix of cancer cells and stroma finding in the HE stained image. c. Cluster 5, which is predominantly TG, show a sparse presence of cancer cells in the stroma in the HE stained image.

We then looked more closely at the pathological findings that corresponded to these clusters. The pathological findings of each cluster are shown in Table 1. This essentially showed that the clusters corresponded to the proportion of cancers and stroma. For example, cancer findings were predominant in clusters 1, 2, and 11, etc., whereas stroma was predominant in clusters 3, 5 and 7, etc. As the next step, we examined lipid molecules expressed in lipidomics-based clusters. The lipid molecules representing each cluster with the highest contribution were identified by the PLS analysis. They included the two main lipid classes (triglycerides (TG) and phosphatidylcholine (PC)), and the three sub lipid classes (phosphatidylethanolamine (PE), sphingomyelin (SM), ceramide (Cer)) (Table 2). Interestingly, clusters diagnosed as C(cancer) in the HE results (Table 1) were composed mainly of PCs, whereas clusters diagnosed as S(stroma) were composed mainly of TG. In addition, clusters diagnosed as C = S were a mixture of PC and TG. Representative findings of the HE-stained images corresponding to each lipid composition are shown in Fig 3. HE findings consistent with cluster 1, which is mainly composed of PC, presenting a dense finding of cancer cells (Fig 3A). HE findings consistent with cluster 4, which is a mixture of PC and TG, show an equal mix of cancer cells and stroma. HE findings consistent with cluster 5, which is predominantly TG, show a sparse presence of cancer cells in the stroma. In addition to PC and TG, PE (cluster 3, 8, 9 and 10), SM (cluster 2) and Cer (cluster 1, 6) were also characteristic lipid classes of the clusters. These clusters were also basically consistent with cancer findings. Furthermore, these lipid classes differed in the length of their side chains and the number of double bonds depending on the cluster. For example, in cluster 1, all PCs tended to have more carbons than the other clusters, with 36 or more carbons. Double bonds also tended to be more than 3, indicating a higher number of double bonds. Conversely, PCs in cluster 13 tended to have a low carbon number of 13. For TG, clusters 3, 4, and 5 had more than 50 carbons, while clusters 6 and 8 had less than 50 carbons. For clusters 7 and 12, molecules reported to be upregulated in breast cancer did not show high PLS coefficients.

**Table 1:** Clusters showing pathological trends with the findings of H&E-stained images.

Cluster no.													
No.	1	2	3	4	5	6	7	8	9	10	11	12	13
**A**	**C**	**C**	**S**	**C = S**	–	–	**S**	–	**C**	–	–	**S**	
**B**	**C**	**C**	**S**	**C = S**	–	–	**S**	–	–	**C**	–	**S**	
**C**	–	**C = S**	**S**	**C = S**	**S**	**C = S**	**S**	**C = S**	**C**	**C**	**C**	**S**	
**D**	**S**	**C**	**S**	**C = S**	–	–	**S**	–	–	**C**	–	**S**	
**E**	–	–	–	**C = S**	**S**	**C = S**	**S**	–	**C**	**C**	–	**S**	
**F**	–	–	**C**	**C = S**	**S**	**C = S**	–	–	**C**	**C**	**C**	**S**	
**G**	–	–	**C**	**C = S**	**S**	**C = S**	–	–	**C**	**C**	–	**S**	**C**
**H**	–	–	–	**C = S**	**S**	–	–	–	–	**C**	–	**S**	
**I**	–	–	–	**C = S**	**S**	–	–	–	–	**C**	**C**	**S**	

C: Cancer, S: stroma. C: The ratio of cancer is higher than that of stroma. S: The ratio of stroma is higher than that of cancer.

**Table 2:** Lipid molecules representing each cluster.

No		HE	PC	TG	PE	SM	Cer
**1**		**C**	**36:3, 38:4, 38:5e,38:6**	**-**	**-**	**-**	**16:0**
**2**		**C**	**34:1**		**-**	**34:1**	**-**
**3**		**S**	**36:5e**	**50:2, 50:3, 52:4**	**38:5e**	**-**	**-**
**4**		**C = S**	**34:1, 34:1e, P-36:2, O-36:3**	**52:4, 54:8**	**-**	**-**	**-**
**5**		**S**	**-**	**50:3, 50:4, 52:4, 52:6, 54:8**	**-**	**-**	**-**
**6**		**C = S**	**36:5, 34:2**	**44:0, 46:0, 46:3, 48:3**	**-**	**-**	**16:0**
**7**		**S**	**-**	**-**	**-**	**-**	**-**
**8**		**C = S**	**32:2, 34:2, 34:4, P-36:2, O-36:3, 36:5**	**48:0, 49:3**	**38:5e**	**-**	**-**
**9**		**C**	**32:0, 32:1e**	**-**	**36:2**	**-**	**-**
**10**		**C**	**36:5e**	**-**	**38:5e**	**-**	**-**
**11**		**C**	**32:1, 34:4**	**-**	**-**	**-**	**-**
**12**		**S**	**-**	**-**	**-**	**-**	**-**
**13**		**C**	**30:1**	**-**	**-**	**-**	**-**

Lipid molecules with high PLS coefficients in each cluster are shown. PC: Phosphatidylcholine, TG: Triglycerides, PE: Phosphatidylethanolamine, SM: Sphingomyelin, Cer: Ceramides. C: Cancer, S: Stroma. C: The ratio of cancer is higher than that of stroma. S: The ratio of stroma is higher than that of cancer.

## Discussion

Heterogeneity of breast cancer is classified into genotype and phenotype [25]. Genotype heterogeneity has been studied using next-generation sequencing [26]. Although gene mutations in breast cancer are less common than in other cancers [27], various genetic tests have been applied in breast cancer. For example, the predictor analysis of microarray 50 (PAM50) test classifies intrinsic subtypes based on 50 genes [1]. The Oncotype DX test measures 21 genes to predict the prognosis and treatment response in luminal breast cancers [2,3]. The measurement of the tumor mutational burden (TMB) can predict the response to immunotherapy based on the amount of genetic mutations [4,28]. Genotype heterogeneity is thus known to be associated with malignancy and prognosis. However, no matter how advanced genomic methods become, the pathological approach to assessing cancer phenotypes will not become unnecessary, but rather increasingly important: phenotypes can reflect the consequences of genomic abnormalities, and it is important to note that such cancer-associated molecular abnormalities may be targets for novel malignancy drugs. Indeed, it has been observed that genotype heterogeneity does not always correlate with phenotype heterogeneity in breast cancer [25]. For example, although a correlation between genotype heterogeneity and prognosis has been reported for HER2-positive breast cancer [29], a study examining phenotype heterogeneity at the protein level with MSI reported an inverse correlation with prognosis [30].

We thus decided to use MSI to examine the phenotype heterogeneity of luminal breast cancer. The luminal breast cancer was indicated to have relatively low genotype heterogeneity in a previous study [1], but the present cluster images clearly show tissue heterogeneity at the lipid molecular level in the luminal breast cancer tissues (Figs 1 and 2). Our finding that phenotype heterogeneity at the lipid molecular level may exist even in luminal breast cancer with few genetic mutations.

The heterogeneity obtained in this study was based on the five lipid classes, i.e., PC, TG, PE, SM, Cer (Table 2). Abnormalities of lipid metabolism in cancer are well known, and a correlation between prognosis and lipids in breast cancer has been noted [30]. PC that composes the cell membrane of cancer cells are different from those of normal cells, since the synthesis of cell membranes occurs actively during cell proliferation. TG serves as a store of fatty acids as ingredient of cell membranes and energy in cancer tissues. PE, while constituting the cell membrane, is also involved in cell motility and cancer cell invasion and metastasis, like PC [31]. SM is the most abundant lipid in the outer layer of the plasma membrane and has been shown to promote cancer development and progression by regulating cell proliferation and migration capacity [30]. SM is degraded to Cer by acid sphingomyelinase (ASM) [32]. In comparison with HE findings, the ratio of PC to TG correlated with the ratio of cancer to stroma. To the best of our knowledge, there are no reports of papers in which the ratio of PC to TG was consistent with the pathological findings of HE-stained images (Fig 3). Based on this, we indicated that the dense areas of cancer cells reflect the abundance of PCs as a result of cancer cell proliferation (Fig 3A). And in areas with sparse cancer cells, the tendency to proliferate is weaker, reflecting the abundance of TG, which stores fatty acids as a source for cell membrane proliferation and energy (Fig 3C). The fact that the clusters with intermediate PC and TG proportions were also consistent with findings of only intermediate cancer and stroma supports these hypotheses (Fig 3B). In addition, not only the ratio of PC to TG, but also the number of carbons and double bonds of the lipid molecules in these lipid classes varied widely. It is known that in cancer, in addition to increased lipid metabolism, elongases [33] that increase the number of carbons and desaturases [5] that increase the number of double bonds are activated. For example, cluster 1 has a high number of carbons and double bonds in the PC and can be considered a cluster that represents highly malignant findings. Cluster 13 can be considered a relatively low malignancy cluster due to the low number of carbons and double bonds in the PC. For TGs, for example, cluster 5 would be underestimated under current assessment criteria because the findings are consistent with stroma and not cancer. However, given the high number of carbons and double bonds in TGs, they could potentially contribute to the malignant potential of cancerous tissue. It is interesting to note that cancer tissues show such a wide diversity in terms of location, suggesting the importance of studying the tissue rather than just the cancer cells.

PE, SM and Cer were also represented in clusters consistent with cancer findings, but no specific findings were found. Therefore, they can be considered to provide information not found in the HE findings. For example, loss of SM has been shown to enhance anti-tumor immunity via Th1 and cytotoxic T cells, and an increased SM/Cer ratio is known to cause cancer immune evasion [31]. Many lipid molecules might be involved in cancer immunity, and the number of reports concerning lipids and cancer immunity have increased in recent years. Among the types of breast cancer, immunotherapy has been applied to the triple-negative type [34], but in a randomized clinical trial of patients with luminal breast cancer, the addition of immunotherapy did not improve progression-free survival or overall survival [35]. It was suggested that this is because luminal breast cancers are associated with lower rates of programmed cell death ligand 1 (PD-L1) positivity [36], lower levels of TILs [37], and a lower median TMB [4]. However, clinical trials of bevacizumab, a humanized anti-VEGF (vascular endothelial growth factor) monoclonal antibody, in combination with immunotherapy are underway in anticipation of bevacizumab’s ability to suppress immune regulation (NCT04732598) [38]. Our present investigation revealed that there is lipidomics-based phenotype tissue heterogeneity even though luminal breast cancer has few gene mutations. If there is a type of luminal breast cancer that responds well to immunotherapy, immune-associated lipids may be related to the treatment response.

### Future perspectives

During this study period, the Oncotype DX genetic test for luminal breast cancer was developed, allowing patient stratification. However, patient stratification in breast cancer remains a challenge, such as stratifying early recurrence groups in triple-negative types. Lehmann classification [39], claudin-low [40] based on genetic analysis contribute to the stratification of triple-negative breast cancer patients. For example, Lehman et al. classified triple-negative breast cancer into 7 subtypes (basal-like 1, basal-like 2, immunomodulatory, mesenchymal, mesenchymal stem-like, luminal androgen receptor, and unstable) and Masuda et al. reported that the pCR rates for neoadjuvant chemotherapy were different between these subtypes [41]. Although they contribute to stratification, they have not yet led to change clinical strategy for breast cancer treatments. We would like to apply the lipidomics-based phenotype heterogeneity assessment method developed in this preliminary study to triple-negative breast cancer to achieve stratification of early recurrence in triple-negative breast cancer, which is considered to have the worst prognosis in the breast cancer field.

## Conclusion

This preliminary study demonstrated that clustering analysis of MSI data can be used to elucidate the lipidomics-based tissue heterogeneity of luminal breast cancer, which is known to have few gene mutations.

## Supporting information

S1 Fig Silhouette plot method. The vertical axis is the silhouette coefficient and the horizontal axis is the number of clusters.
(PPTX)

S2 Fig Representative examples of clusters showing pathological trends. a. The *white dotted line* corresponds to the area of cluster 1. Cluster 1 corresponds to the cancer findings in the H&E images. The *black dotted line* corresponds to the area of cluster 7. Cluster 7 corresponds to the stromal findings in the H&E images. b. The *white dotted line* corresponds to the area of cluster 2. Cluster 2 corresponds to the cancer findings in the H&E images. The *black dotted line* corresponds to the area of cluster 3. Cluster 3 corresponds to the stromal findings in the H&E images.
(PPTX)
