# Supplementary figures and images for: Lipidomics-based tissue heterogeneity in specimens of luminal breast cancer revealed by clustering analysis of mass spectrometry imaging: A preliminary study
Source: PLoS One. 2023 May 10;18(5):e0283155. doi: 10.1371/journal.pone.0283155 (PMC10171676; doi:10.1371/journal.pone.0283155)

## Slide 1
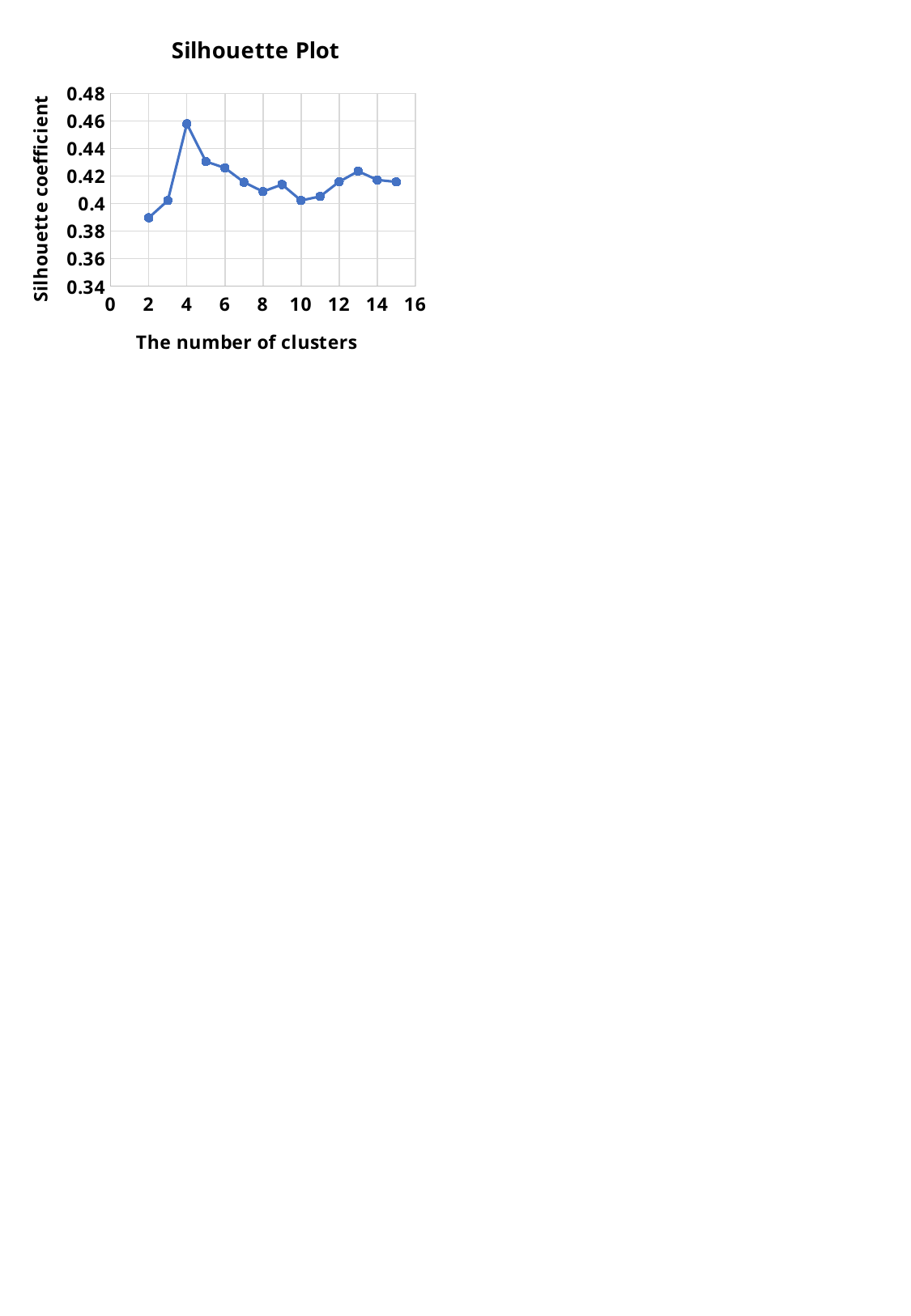

### Chart: Silhouette Plot
| Category | |
|---|---|

Supplement: S1 Fig — The vertical axis is the silhouette coefficient and the horizontal axis is the number of clusters. (PPTX) [file pone.0283155.s001.pptx]

## Slide 1
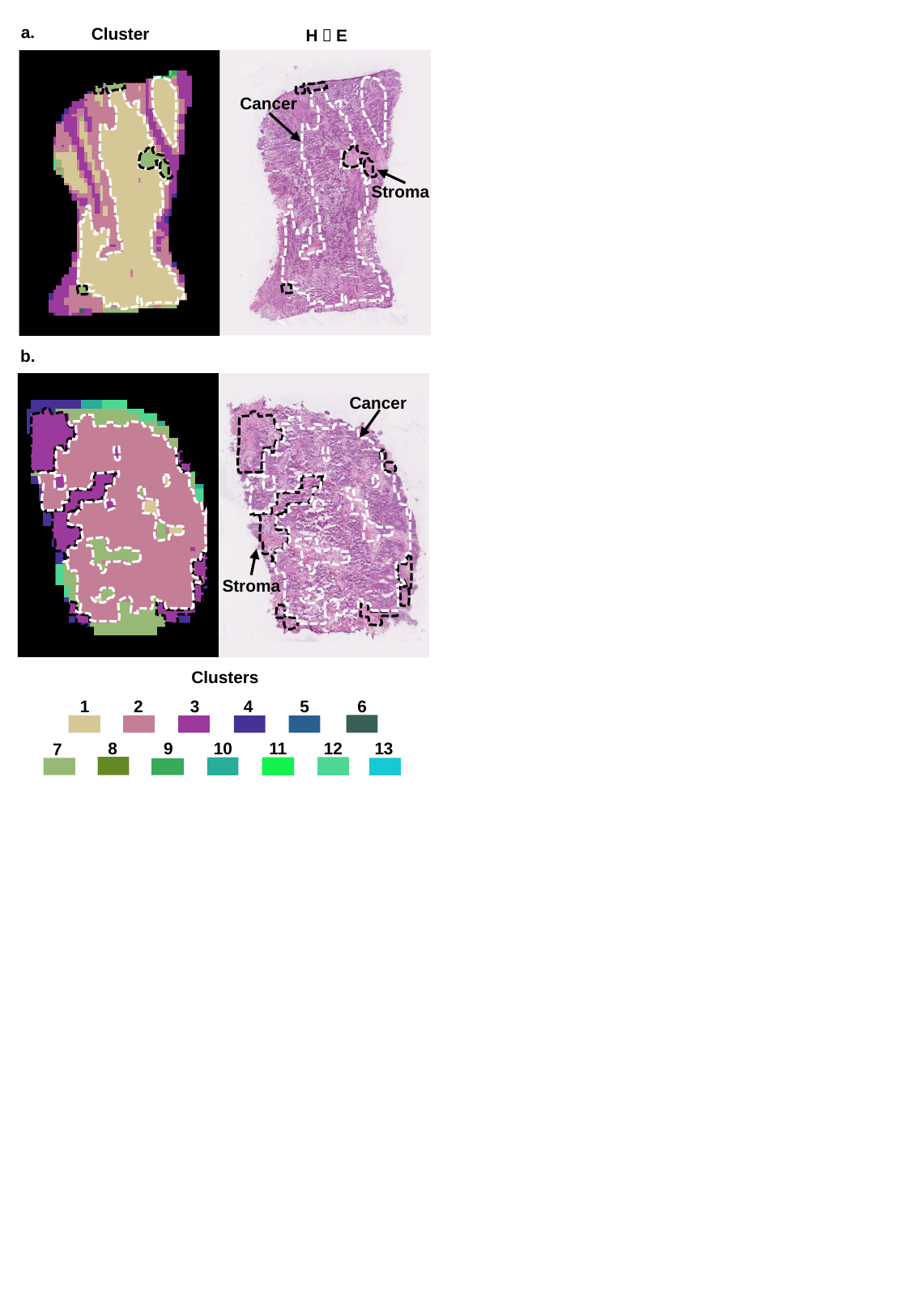

a.
Cluster
H＆E
Cancer
Stroma
b.
Cancer
Stroma
Clusters
1
2
3
4
5
6
12
10
8
9
11
13
7

Supplement: S2 Fig — a. The white dotted line corresponds to the area of cluster 1. Cluster 1 corresponds to the cancer findings in the H&E images. The black dotted line corresponds to the area of cluster 7. Cluster 7 corresponds to the stromal findings in the H&E images. b. The white dotted line corresponds to the area of cluster 2. Cluster 2 corresponds to the cancer findings in the H&E images. The black dotted line corresponds to the area of cluster 3. Cluster 3 corresponds to the stromal findings in the H&E images. (PPTX) [file pone.0283155.s002.pptx]
